# The Application of *Citrus folium* in Breast Cancer and the Mechanism of Its Main Component Nobiletin: A Systematic Review

**DOI:** 10.1155/2021/2847466

**Published:** 2021-06-29

**Authors:** Yuan Wu, Chien-shan Cheng, Qiong Li, Jing-xian Chen, Ling-ling Lv, Jia-yue Xu, Kai-yuan Zhang, Lan Zheng

**Affiliations:** ^1^Department of Traditional Chinese Medicine, Shanghai Jiao Tong University School of Medicine Affiliated Ruijin Hospital, Shanghai 200025, China; ^2^Department of Integrative Oncology, Fudan University Shanghai Cancer Center, Shanghai 200032, China; ^3^Department of Oncology, Shanghai Medical College, Fudan University, Shanghai 200032, China

## Abstract

*Citrus folium* and its main ingredient nobiletin (NOB) have received widespread attention in recent years due to their antitumor effects. The antitumor effect of *Citrus folium* is related to the traditional use, mainly in its Chinese medicinal properties of soothing the liver and promoting qi, resolving phlegm, and dispelling stagnation. Some studies have proved that *Citrus folium* and NOB are more effective for triple-negative breast cancer (TNBC), which is related to the syndrome of stagnation of liver qi. From the perspective of modern biomedical research, NOB has anticancer effects. Its potential molecular mechanisms include inhibition of the cell cycle, induction of apoptosis, and inhibition of angiogenesis, invasion, and migration. *Citrus folium* and NOB can also reduce the side effects of chemotherapy drugs and reverse multidrug resistance (MDR). However, more research studies are needed to clarify the underlying mechanisms. The modern evidence of *Citrus folium* and NOB in breast cancer treatment has a strong connection with the traditional concepts and laws of applying *Citrus folium* in Chinese medicine (CM). As a low-toxic anticancer drug candidate, NOB and its structural changes, *Citrus folium*, and compound prescriptions will attract scientists to use advanced technologies such as genomics, proteomics, and metabolomics to study its potential anticancer effects and mechanisms. On the contrary, there are relatively few studies on the anticancer effects of *Citrus folium* and NOB *in vivo*. The clinical application of *Citrus folium* and NOB as new cancer treatment drugs requires *in vivo* verification and further anticancer mechanism research. This review aims to provide reference for the treatment of breast cancer by Chinese medicine.

## 1. Introduction

According to the latest data from the International Agency for Research on Cancer (IARC) in 2018, the incidence of breast cancer (BC) in female cancers worldwide is 24.2%, ranking first among female cancers, which is the main cause of female cancer deaths [1]. In the early stage, symptoms such as breast lumps, nipple discharge, and axillary lymphadenopathy are often manifested. In the late stage, cancer cells may metastasize to a distance, and multiple organ diseases may appear, which directly threaten the life of the patient. Although radiotherapy, chemotherapy [2], and immunotherapy [3] can inhibit the further growth and proliferation of tumors to achieve the purpose of suppressing tumors, they have large toxic side effects, such as bone marrow suppression [4], allergic symptoms [5], skin-related symptoms [6], and gastrointestinal distress [7]. There are still certain limitations in postoperative tumor recurrence, bone metastasis, and multidrug resistance (MDR) [8–10]. Therefore, it is very important to find a way to reduce toxic side effects and increase sensitivity.

As one of the main complementary and alternative medicines, traditional Chinese medicine (TCM) has a history of thousands of years [11]. It has the advantages of multiple targets, small side effects, and significant clinical effects in cancer treatment [12–15]. Drug screening from natural medicines is a feasible shortcut, such as paclitaxel extracted from *Taxus mairei* [16], Kanglaite injection extracted from *Coix lachryma-jobi semen* [17], and Cinobufacini extracted from *Toad skin cutis bufonis* [18]. We have discovered from the compound prescriptions that *Citrus folium* is commonly used to treat BC as Chinese medicine (CM). *Citrus folium* is derived from the leaves of the Rutaceae plant Tangerine and its cultivated varieties. It has the effects of soothing the liver, promoting qi, removing phlegm, and resolving mass. Modern pharmacological studies have shown that *Citrus folium* mainly contains volatile oil, carbohydrates, flavonoids, and other ingredients, which can effectively treat cough, pulmonary carbuncle, breast carbuncle, hypochondriac pain, abdominal distension, hernia, edema, etc. [19]. However, the material basis of its efficacy is still unclear, so the mechanism of BC treatment at the molecular level is worthy of further study.

**Figure 1:**
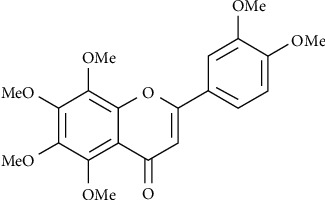
The chemical construction of NOB.

NOB is one of the main components of *Citrus folium*, with a content of about 1.94% [20], which is essentially polymethoxyflavones (PMFs) [21]. Because of its more methoxy groups, it has high-fat solubility. The molecular formula is C_21_H_22_O_18_, and the weight is 402.399 g/mol. Its structure is shown in Figure 1. In addition to separating NOB from *Citrus folium*, there is also a small amount of NOB in *Citri reticulate pericarpium* [22]. Experimental studies *in vivo* and *in vitro* have confirmed that NOB has a wide range of effects, including anti-inflammatory [23–25], antiviral [26, 27], inhibition of oxidative stress [27], antiatherosclerosis [28], nerve protection [29–31], antidementia [32, 33], alleviation of ischemia-reperfusion injury [34, 35], and antidiabetic [36]. Not only that, NOB is also quite significant in the tumor treatment, including nasopharyngeal cancer [37], breast cancer [38], lung cancer [39], gastric cancer [40], colorectal cancer [41], liver cancer [42], prostate cancer [43], kidney cancer [44], bladder cancer [45], ovarian cancer [46], glioma [47], osteosarcoma [48], and other different types. It can inhibit tumor cell growth and proliferation [49], can inhibit invasion and migration by inhibiting EMT [39, 50] and angiogenesis [51], regulates the tumor cell cycle to block it in the *G*1 phase [51], and can induce apoptosis, which can be used to “precise” the treatment of tumors in terms of tumorigenesis, promotion, and prognosis. Since the source of NOB is *Citrus folium*, which is often used to treat BC in CM, we decided to study the mechanisms of NOB in the BC treatment.

Based on the combination of prescriptions and syndromes, we found that *Citrus folium* is mostly used to treat BC. Therefore, we review the traditional use history of *Citrus folium*, from the clinical application of *Citrus folium* to the pharmacological research of NOB, focusing on the anti-BC effects and their prospects as new antitumor drugs.

## 2. Research Methodology

This review was conducted following the Preferred Reporting Items for Systematic Reviews and Meta-Analyses (PRISMA) statement [52].

### 2.1. Search Strategy

Every publication in English that was reviewed for this study was extracted from the PubMed, Embase, Web of Science, and CNKI databases restricted to the Medical Subject Headings Index (MeSH/DeCS) to April 2021. The search was based on different combinations of the following keywords: “*Citrus folium*,” “*Folium citrus reticulate*,” “nobiletin,” “NOB,” “mechanism,” “anti-breast-cancer,” and “pharmacology.” Furthermore, we reviewed the references in the selected articles for additional reports not included in the original article search.

### 2.2. Study Selection

**Figure 2:**
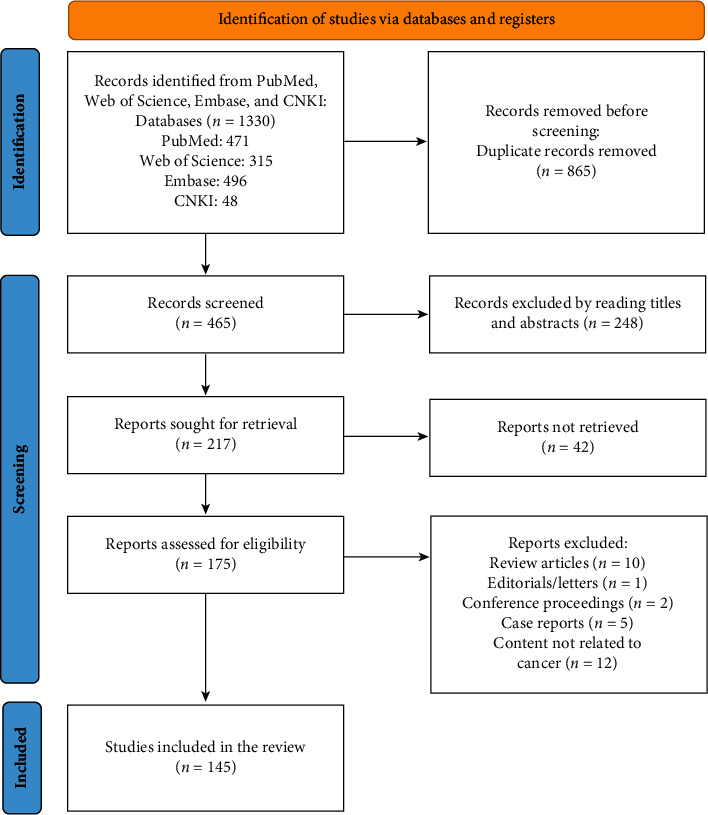
The flow of the literature search.

The two authors independently extracted and proofread the titles and abstracts of each article. The inclusion criteria were the mechanism of *Citrus folium* and NOB in the treatment of breast cancer, including *in vivo* and *in vitro* models. The authors excluded articles based on the following criteria: review articles, abstracts, editorials/letters, conference proceedings, case reports, and the content not related to cancer. Differences of opinion between two authors were determined independently by the third author. The flow of the literature search is presented in Figure 2.

## 3. Results

### 3.1. TCM Understanding of the Etiology and Pathogenesis of BC

#### 3.1.1. Ancient Chinese Medicine's Views on BC

In different historical periods, Chinese physicians have different understandings of the etiology and pathogenesis of BC. During the Jin, Sui, and Tang Dynasties, most physicians believed that the tumorigenesis and progression of BC were related to qi stagnation and blood stasis; during the Song, Jin, and Yuan Dynasties, physicians emphasized the effects of internal emotional injuries, the liver-spleen disharmony, and qi-blood deficiency; in the Ming and Qing Dynasties, the etiology and pathogenesis of BC were mainly liver dysfunction, lack of kidney qi, and imbalance of thoroughfare vessel (Chong channels) and conception vessel (Ren channels) [53]. We found that the understanding of BC in different historical periods has one thing in common: stagnation of liver qi, indicating that qi stagnation is the main cause of most breast cancers.

For example, for the pathogenesis of BC, Chen Shigong described it in detail in *Orthodox Manual of External Medicine* (Wai Ke Zheng Zong, Ming Dynasty, AD 1617) [54]: “*the etiology of BC is that depression causes liver-spleen disharmony, then the viscera is weak, which eventually leads to breast lumps like stones (Ru Yan)*.” In *Collected Works of Materia Medica* (Ben Cao Hui Yan, Ming Dynasty, AD 1624), the etiology of breast tumors is described: “*blood-qi stagnation, and the accumulation of phlegm and saliva, lead to hypochondriac pain, mammary carbuncle, swelling toxin and other diseases* [55].” According to the theory of TCM, the main reason for the formation of BC is an emotional injury such as depression and anxiety and visceral dysfunction which causes phlegm accumulation and blood stasis in the breast, eventually leading to breast cancer. Therefore, the initial cause of BC is emotional injury.

#### 3.1.2. Modern Chinese Medicine's Views on BC

Modern Chinese medicine also believes BC is closely related to qi and blood. According to the classification of BC syndromes in the “Standards of Chinese Society of Chinese Medicine/Guidelines for the Diagnosis and Treatment of Tumors in TCM,” 2008 edition [56], they are divided into the stagnation of liver-qi, accumulation of heat-induced toxicity, thoroughfare vessel (Chong channels) and conception vessel (Ren channels) disorder, deficiency of both qi and blood, spleen and stomach weakness, and yin deficiency of the liver and kidney. Through the analysis of the BC research literature, we summarize the distribution characteristics of its common syndromes and general symptoms (as shown in Table 1). With the tumorigenesis, promotion, and metastasis of BC, the main syndrome differentiation types in each process are different. The early stage is mostly qi stagnation and blood stasis, accumulation of phlegm and dampness, and heat-induced toxicity [57]. In the later stage and after metastasis, they are mostly yin deficiency of the liver and kidney, yang deficiency of the spleen and kidney [58], and vital qi insufficiency.

#### 3.1.3. The Connection between TCM and Western Medicine about the Tumorigenesis of BC

The essence of tumorigenesis is gene mutation, which is closely related to the constitution of TCM [59]. Certain constitution such as deficiency of vital qi are high risk factors for BC [60]. The study found that the BRCA gene mutation rate of TNBC was 11.2% [59]. Professor Wan believes that it is consistent with the concept of congenital deficiency in TCM [61].

Negative emotions, as a stressor of the human body, affect its endocrine, nervous, and immune systems and increase the rate of recurrence and metastasis of BC [62]. By activating the renin-angiotensin system, it promotes the secretion of adrenal corticosterone, reduces the activity of NK cells, and ultimately weakens the patient's immune supervision ability [63]. This is the same as the etiology and pathogenesis of the “stagnation of liver qi” in TCM.

Modern medicine believes that increased intake of high-fat diet is related to the incidence of BC. It has been reported that long-term intake of high fat will increase the risk of BC and show a certain dose effect [64]. There is also evidence that Asian postmenopausal women who prefer “meat-sweet food” diets have a ratio of 1.3 associated with BC risk [65]. Phlegm stagnation caused by unregulated diet is indeed related to the microenvironment of BC. In breast tumors, adipose tissue occupies a large proportion in the interstitium, forming a unique tumor-associated adipose tissue (CAAT), which is the fat microenvironment around the tumor [66]. Adipocytes can abnormally secrete tumor-related adipokines and a large number of metabolic substrates, providing energy for tumor cell proliferation and migration [67]. Adipokines and metabolic substrates are the “turbid toxins” of TCM, which are difficult to transfer normally, and can be transformed into lumps [68]. They are not only a pathogenic factor of BC but also provide a material basis for the tumor microenvironment.

Breast cancer is prone to lung metastasis and brain metastasis [69, 70]. This is closely related to the stagnation of qi due to phlegm, phlegm-dampness obstructing the lung, and the orifices confused by phlegm. It also shows the process of phlegm syndrome from shallow to deep, from light to severe. In addition, bone metastasis is related to the disharmony between nutritive qi and defensive qi. The ability of blood to nourish bones and defensive qi to resist exogenous pathogens decreases, causing BC cytokines to interact with cells in the bone microenvironment, thereby triggering bone metastasis [71].

Based on the high-risk factors of breast cancer, we tried to explain the similarities between the etiology and pathogenesis of TCM and the pathophysiology of Western medicine in the tumorigenesis.

It can be seen from Table 1 that the pulses of all syndrome types are wiry and thread pulses (Xi and Xian Mai), indicating that the tumorigenesis of the breast is related to the stagnation of liver qi. *Citrus folium* can promote qi and dispel stagnation, so it is mainly used to treat the qi stagnation type of BC. However, the combination of disease-syndrome-prescriptions and molecular research is still lacking, so we summarized the application of *Citrus folium* in different prescriptions and the molecular mechanism of its main component NOB.

### 3.2. The Application of *Citrus folium* in the Treatment of BC in TCM

The record of *Citrus folium* can be traced back to *Addendum to the Derivation of Herbs* (Ben Cao Yan Yi Bu Yi, Yuan Dynasty, AD 1347) compiled by Zhu Danxi. The description of the nature and flavor of *Citrus folium* is in *Compendium of Materia Medica* (Ben Cao Gang Mu, Ming Dynasty, AD 1596) [72]. The large-scale use of *Citrus folium* was in the Ming and Qing Dynasties, such as Bamboo women's region (Zhu Lin Nv Ke, Qing Dynasty, AD 1817), wrote by the famous CM physician, Ye Tianshi, which pointed out that *Citrus folium* can be used to treat breast cancer (*Ru Yan*) [73]. In addition to the traditional oral form, *Citrus folium* can also be used externally. *Citrus folium* has many traditional formulas for treating BC (as shown in Table 2), and most of the corresponding syndromes are liver-qi stagnation.

Most of the modern Chinese patent medicine preparations for the treatment of breast lumps contain *Citrus folium*, which is made into tablets, capsules, powders, liquid preparations, etc., including *Rukuaixiao tablet*, *Rupikang tablet*, and *Lirukang capsule* [78]. Most of the clinical empirical prescriptions of modern Chinese medicine for treating BC contain *Citrus folium*, such as prescription for regulating the liver and tonifying the kidney in our team [79]. However, TCM is not clear about the pathological nature of the disease, and the specific mechanism of *Citrus folium* in the treatment of BC needs to be studied on its active ingredient.

### 3.3. Extraction Methods and Mechanisms of NOB

#### 3.3.1. NOB Extraction Methods

To clarify the specific pathways for the effective components in *Citrus folium* to treat tumors, the researchers carried out alcohol extraction, chromatography, crystallization, etc., on *Citrus folium* and successfully separated and purified NOB [80, 81]. Dong et al. used 95% ethanol to immerse and reflux the *Citrus folium* coarse powder, then used small-pore resin (MCI) column chromatography to preliminarily separate the ethanol extract, and finally purified NOB by silica gel, MCI, ODS, polyamide, Sephadex LH-20, and other column spectrum analyses [20]. There are usually many methods for extracting NOB. The most commonly used is the petroleum ether [82] solvent extraction method. It can also be extracted with acetone, absolute ethanol, methanol, ethyl acetate, etc. And ultrasonic-assisted extraction can be used to improve efficiency [83] by destroying the cell membrane and accelerating cell lysis [84]. Chromatography can identify and quantitatively analyze flavonoids, such as column chromatography [85] and high-speed countercurrent chromatography [86, 87].

#### 3.3.2. Nobiletin's Mechanisms on Different Conduction Pathways in BC

**Figure 3:**
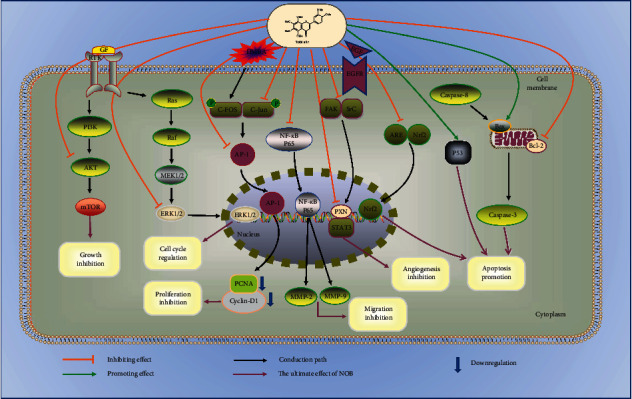
The antitumor mechanism of NOB in BC.

NOB plays a certain role in the BC treatment in many aspects, such as inhibition of tumor cell growth and proliferation, regulation of the tumor cell cycle, induction of tumor cell apoptosis, regulation of tumor-related protein expression, inhibition of migration and invasion, and inhibition of angiogenesis. The specific mechanisms and conduction pathways are summarized in Figure 3 and Table 3.

From Table 3, we can conclude that NOB can inhibit the phosphorylation of AKT and downstream mTOR in MDA-MB-468 cells but does not inhibit the activity of these two molecules in other BC cell lines. Bcl and Bax were only involved in the apoptosis of MDA cells, but not in MCF-7 and SK-BR-3 cells [94]. It indicates that NOB may be more effective on the MDA-MB-468 cell line, which belongs to TNBC. In other words, NOB might be more effective for TNBC.

*(1) Tumor Cell Growth Inhibition*. The primary difference between tumor cells and normal cells is that the growth rate is greatly accelerated, and the number of tumor cells increases sharply in a short period. NOB showed a dose-dependent growth inhibitory activity on three types of BC cells. The largest growth inhibitory effect was observed in MDA-MB-468, while in SK-BR-3, the lowest growth inhibitory effect was observed [94]. Studies have shown that NOB can inhibit the growth of TNBC's MDA-MB-468 cells by inhibiting the AKT-mTOR pathway [94]. Furthermore, NOB can significantly reduce MCF-7 cell viability and inhibit cell growth in a dose-dependent manner [88]. The reason is that NOB induces its metabolism by upregulating cytochrome P450-1A1 (CYP1A1) and cytochrome P450-1B1 (CYP1B1), thereby enhancing its cytostatic effect in MCF-7 BC cells [90]. Experiments have proved that NOB reduces the number of Hs578T cells [89]. All these indicate that NOB can inhibit the growth of BC cells.

*(2) Tumor Cell Proliferation Inhibition*. Cell proliferation is regulated by genes. c-Fos and c-Jun form a dimer, forming transcription factor AP-1 [95]. The transcription and protein expression of genes such as AP-1, cyclin D1, and PCNA [96] can promote cell proliferation [97, 98]. NOB can bind c-Jun competitively to prevent it from forming dimers, inhibit AP-1 from entering the nucleus, and then reduce the expression of PCNA and cyclin D1, thereby inhibiting proliferation. This shows that NOB inhibits cell proliferation through the AP-1 signaling pathway [93]. Studies have confirmed that the survival rate and proliferation ability of MCF-7 cells decreased after NOB treatment, and the cell morphology changed from diamond to round [88]. These suggest that NOB can inhibit tumor cell proliferation.

*(3) Tumor Cell Cycle Regulation*. A variety of experiments and clinical studies have found that cell cycle regulation disorder is one of the characteristics of tumors and suggest that tumorigenesis may be the result of abnormal cycle regulation [89]. The cell cycle regulation mechanism is under the precise regulation of related genes and runs according to certain rules and rhythms, which determines the growth, division, and death of cells. The abnormality of the cycle regulation can lead to the continuous proliferation of cells, which is the biological basis of tumorigenesis.

NOB regulates the cycle differently for different types of BC cells, such as hormone receptor-positive MCF-7, HER2-positive SK-BR-3, and triple-negative MDA-MB-468 cells. NOB induces MDA-MB-468 cell cycle arrest in the *G*0/*G*1 phase by inhibiting ERK1/2 activity [94], and MCF-7 cells also have significant accumulation in the *G*1 phase, but for SK-BR-3 cells, the proportion of cells in each cell cycle stage did not change significantly. NOB can increase the *G*2/*M* phase arrest of Hs578T cells by 14.3% [89]. Cyclin-D1 is a key regulator of the *G*0/*G*1 cell cycle checkpoint. NOB inhibits the *G*0/*G*1 cell cycle by reducing the expression of cyclin-D1 and simultaneously upregulating p21 [94].

*(4) Tumor Cell Apoptosis Induction*. In multicellular organisms, the balance between cell death and growth is very important for the development and maintenance of body functions. Disorders of balance can lead to the progression of cancer. Therefore, the balance must be strictly controlled, and the presence of interference can be eliminated through a process called programmed cell death [99]. Apoptosis occurs in two main ways: one is the exogenous way and the other is the endogenous way. The external pathway is through ligand binding to activate the death receptor leading to the activation of caspase-8, while the internal pathway is through the mitochondrial pathway [99].

Studies have shown that the cells after NOB treatment have apoptotic morphological changes in the nucleus, including apoptotic bodies and nuclear condensation [88]. NOB can activate Bax (proapoptotic effect) in MCF-7 cells, then increase the permeability of the outer mitochondrial membrane, and release cytochrome C (Cyt-c) from the mitochondria into the cytoplasm, leading to the activation of the apoptotic complex [100]. On the contrary, NOB can inhibit Bcl-2, which has the effect of inhibiting apoptosis [101]. After Bcl-2 is reduced, the intracellular cascade activity is activated, leading to cell apoptosis [102, 103]. In addition, NOB increases the phosphorylation of p38 and reduces the transport of p65 and Nrf2 to the nucleus [88]. This shows that NOB can induce BC cell apoptosis from multiple angles and pathways.

*(5) Migration and Invasion Inhibition*. Metastasis of the tumor involves local invasion and blood diffusion. The adhesion between tumor cells mediated by cell adhesion molecules is reduced. Tumor cells adhere closely to the basement membrane and secrete proteolytic enzymes to degrade the extracellular matrix, causing local defects in the basement membrane. After the cancer cells move through the dissolving basement membrane defect with amoeba, they continue to dissolve the interstitial connective tissue and move in the interstitium. When they reach the blood vessel wall, they pass through the basement membrane of the blood vessel and enter the blood vessel in the same way, forming the new metastases eventually [104].

MMPs participate in the degradation of the basement membrane and are a necessary process for migration [104]. NOB can downregulate the protein expression of MMP-2 and MMP-9 at the transcription and translation levels and then inhibit cell migration [88]. Moreover, NOB can change the morphology of MCF-7 cells, making them to shrink and weakening their adhesion [88]. The results of the scratch test on MCF-7 cells showed that the inhibitory effect of NOB on the stenosis of the defect area was dose dependent [88]. Borah et al. also proved that NOB significantly reduces the migration rate of Hs578T cells [89]. And NOB also reduces the invasion behavior of MDA-MB-231 cells [38]. These experiments showed that NOB can inhibit the migration and invasion of a variety of BC cells.

*(6) Angiogenesis Inhibition*. Tumor angiogenesis is considered to be a key sign of tumor progression. The formation of new blood vessels is to make up for the lack of blood supply and provide sufficient oxygen for tumor progression [105]. Vascular endothelial growth factor (VEGF) is the main growth factor in tumor angiogenesis, while NOB can inhibit angiogenesis by inhibiting VEGF. NOB can also directly dock with the ATP-binding site of EGFR, which has an inhibitory effect. NOB can competitively bind to the site (GAS element, ttctgggaa) where STAT3 initiates transcription, to inhibit the expression level of the STAT3/PXN complex, thereby inhibiting angiogenesis at the transcriptional level [91].

In short, NOB plays a certain role in the BC treatment in many aspects, such as inhibition of tumor cell growth and proliferation, regulation of the tumor cell cycle, induction of tumor cell apoptosis, inhibition of migration and invasion, and inhibition of angiogenesis. However, most of the experiments are *in vitro*; perhaps, we could confirm its effects *in vivo* in the future.

#### 3.3.3. NOB Reducing Chemotherapeutic Drugs' Toxicity and Reversing MDR

*(1) Reducing the Toxicity of Chemotherapeutic Drugs*. NOB can improve the efficacy of chemotherapeutic drugs and reduce the dosage [106]. For example, doxorubicin is a commonly used chemotherapy drug in the BC treatment. This drug's use effect is low, and it is easy to produce toxic to normal tissues [107]. Studies have shown that NOB can not only increase the cytotoxic activity of doxorubicin on MCF-7 cells but also inhibit the overexpression of adhesion molecules in vascular endothelial cells caused by doxorubicin, thereby reducing cardiotoxicity [106]. NOB also increased the chemotherapeutic sensitivity of BC cell MDA-MB-231 to epirubicin by regulating PER2. It shows that NOB can not only reduce the toxicity of chemotherapy drugs but also increase the effect of chemotherapy drugs.

*(2) Reversing Multidrug Resistance*. The mechanisms of MDR are the overexpression of ATP-binding cassette (ABC) membrane transporters, especially P-gp, which is considered to be the main factor.

Scientists found that NOB not only increased the accumulation of chemotherapy drugs in ABCB1-overexpressed cancer cells by inhibiting p-gp but also inhibited the Nrf2/Akt/ERK pathway, thereby enhancing the anticancer effect of paclitaxel in drug-resistant cancer cells [108]. NOB can also reverse drug resistance of PTX in MDR tumors by inhibiting the Akt/ERK/NRF2 pathway [109]. Therefore, NOB can enhance chemotherapy drugs' effects by alleviating MDR.

#### 3.3.4. Pharmacodynamics and Pharmacokinetics of NOB

Recently, some reports have proposed that NOB is absorbed by proton-linked carboxylic acid transporters in the intestinal tract of Caco-2 cells [110]. Caco-2 cells are similar in morphology and function to small intestinal epithelial cells and are widely used to study *in vitro* models of drug absorption. The results show that the absorption of NOB is saturated at low concentration and unsaturated at high concentration [111]. The absorption of NOB by Caco-2 cells is mainly mediated by passive diffusion [111].

Studies have shown that NOB significantly accumulates in the blood, urine, stomach, small intestine, liver, and kidney within 1 to 4 hours of administration. In addition, a large amount of NOB remained in the gastric muscle layer 1 hour after administration, indicating that NOB may enter the circulation through the gastric muscle layer [112], probably due to the high hydrophobicity of NOB [113].

After oral intake, NOB further forms a variety of metabolites [114]. NOB generally undergoes phase I and phase II metabolism, and more than 16 metabolites have been identified so far [115]. After NOB is metabolized by mice, the three common phase I metabolites in urine are 3′-DMN, 4′-DMN, and 3′,4′-DMN [116]. According to some reports, the metabolism of flavonoids in animals is carried out by hydroxylation and oxidative demethylation of cytochrome p450 [117]. Furthermore, *in vivo* experiments have shown that the phase I demethylation of NOB is caused by cytochrome P450. Researchers found that CYP1A1, CYP1A2, and CYP1B1 have high activity in the formation of 3′-DMN, confirming that these enzymes are involved in the metabolism of NOB [118].

NOB undergoes phase II metabolism in the small intestine [119]. Phase II metabolites contain glucuronic and sulfonic conjugates in the serum, bile, and urine of rats [119]. The unabsorbed compounds in the small intestine will enter the large intestine. After being decomposed by the intestinal microorganisms, some of them will be reingested, and the others will enter blood [120].

The intestinal efflux transporter (P-gp), which is widely distributed in the mucosa of intestinal epithelial cells, can pump the absorbed NOB out of the cell membrane and return to the intestinal lumen; it can also cooperate with metabolic enzymes to prevent the absorption of NOB. Eventually, NOB is excreted through urine or feces [121].

NOB has been reported to be a safe agent for *in vivo* use as it has not caused any long-term toxicity or significant weight and liver weight reduction, which are the signs of toxicity [27, 93, 122, 123]. Because of the existence of a large number of methoxy groups and high hydrophobicity, NOB has poor water solubility (less than 1 *μ*g/ml) and low bioavailability [124]. And it is easy to crystallize in the human body at room temperature and is difficult to absorb [125]. Therefore, to improve its bioavailability, researchers have used ASDs (amorphous solid dispersions) to improve the solubility and gastrointestinal absorption of NOB to maintain drug concentration by increasing dissolution rate and preventing recrystallization [126]. And nanotechnology can be used to give NOB chemical stability to improve its bioavailability and therapeutic effect [127].

## 4. Discussion

Most of the current studies are that NOB directly acts on tumor cells. The mechanisms contain inhibiting cell growth and proliferation, regulating the cell cycle, promoting cell apoptosis, and inhibiting migration and invasion from the level of genes, transcription, and translation. However, the effects of NOB on metabolism, immune cells, intercellular substances, and tumor microenvironment need to be further studied. Most of the experiments of NOB are cell experiments, and there is only one *in vivo*. Since the tumor microenvironment is relatively complex, experiments *in vivo* are more accurate and convincing. In the future, it is necessary to further study whether the role of NOB *in vivo* is consistent with that *in vitro* and whether there is any interaction with other tissues. In addition to nude mice, other animal models can be tried, such as immune survivable mice and zebrafish. Zebrafish is also a well-developed ideal *in vivo* model. For metastatic TNBC, it can simulate the process of metastasis in a short period of time, and the detection is relatively simple. In addition, there are relatively few studies on NOB combined with chemotherapy and targeted drugs in the treatment of BC. Experiments should be carried out to observe the effects of NOB combined with other treatments.

NOB is highly fat-soluble, while traditional Chinese medicine decoctions are mostly water decoctions. The possible reason is the dosage of *Citrus folium* or the compatibility of CM having an impact on its efficacy or other ingredients in *Citrus folium* making effects, which needs further study. The bioavailability of NOB is low. To increase the bioavailability, we can try to change the dosage form, such as intravenous administration, or make structural modifications or change the biological activity of NOB through nanoadsorbed drugs. The biological activities of the metabolites of NOB entering the body, 3′-DMN, 4′-DMN, and 3′,4′-DMN, are also worth exploring. Different effects of *Citrus folium* on different targets of ER, PR, and HER2 also require clinical research studies.

Discovering the antitumor mechanism of NOB has realized the research from clinical to basic and then from basic to clinical, which has certain potential value. From a molecular perspective, compared with other cell lines, NOB shows more cell proliferation inhibitory effects on the TNBC cell line MDA-MB-231, indicating that NOB may be more effective on TNBC. From the perspective of TCM syndrome differentiation, receptor-positive patients are usually treated with endocrine drugs and are mostly of Yin deficiency and heat, while TNBC patients are mostly of qi stagnation and blood stasis type. *Citrus folium* enters the liver meridian, which has the effect of regulating qi and dispelling stagnation. It may be more effective for TNBC patients. In summary, it is possible to further screen the more effective syndromes of NOB treatment, with a view to the more precise treatment of different types of BC.

## 5. Conclusion

A majority of studies focusing on the anticancer effects of *Citrus folium* and its main active ingredients have been carried out and identified in the past ten years. Although the clinical application of *Citrus folium* in TCM has a long history, its specific mechanism deserves further exploration, such as its role in multiple system diseases and its future application as an anticancer molecular target. The precise molecular action of each identified compound and biological activities of multicompounds remains to be elucidated. Therefore, it is necessary to study the effects of *Citrus folium* and NOB *in vivo* through a variety of applications, such as topical, oral, or injection. Further research warrants investigation into the effect of *Citrus folium* and its active components on the tumor-related immune functions, cancer stem cells, and the tumor microenvironment to provide more throughout picture of its action mechanism for cancer therapies.

## Figures and Tables

**Table 1:** The syndrome of BC in TCM.

Syndromes	Symptoms	Tongue and pulse	Main symptoms	Stage
Disharmony of the thoroughfare vessel (Chong channels) and conception vessel (Ren channels)	Menstrual changes, lassitude, tidal fever and night sweating, soreness and weakness of the waist and knees, irritability, dry mouth, anorexia, dizziness	Light color of the tongue, thin fur, wiry pulse, thread pulse	Menstrual changes, tidal fever and night sweating, dry mouth	I

Stagnation of liver qi	Depression, hypochondrium distending pain, soreness and weakness of the waist and knees, menstrual changes	Reddish tongue, thin fur, wiry pulse, thread pulse	Hypochondrium distending pain, depression	I

Vital qi insufficiency and heat flourishing	Irritability, menstrual changes, lassitude, anorexia, soreness and weakness of the waist and knees, insomnia	Light color of the tongue, yellow and greasy fur, wiry pulse, thread pulse, weak pulse	Lassitude, tidal fever, yellow fur	II

Qi and Yin deficiency	Lassitude, depression, menstrual changes, anorexia, tidal fever and night sweating, dry and itchy throat, insomnia	Light color of the tongue, wiry pulse, thread pulse	Lassitude, tidal fever, and night sweating	III IV

Yin deficiency of the liver and kidney	Lassitude, soreness, and weakness of the waist and knees, insomnia, dizziness, dry and itchy throat, tidal fever and night sweating, tinnitus, forgetfulness	Reddish tongue, thin fur, wiry pulse, thread pulse	Soreness and weakness of the waist and knees, insomnia, tidal fever, tinnitus	III IV

**Table 2:** The summary of *Citrus folium* treating breast-related diseases.

Source	Prescription	Composition	Name of the disease	Syndrome type	References
Experience Gained in Treating External Diseases (Yang Ke Xin de Ji, Qing Dynasty, AD 1805)	*Citrus folium* decoction	*Citrus folium, Taraxaci cum radice herba, Fritillariae thunberii bulbus, Prunellae vulgaris spica, Citri reticulatae viride pericarpium, Angelicae sinensis corpus Radicis, Paeoniae rubrae radix, Trichosanthis kirilowii radix, Cyperi rotundi rhizoma, Scutellariae baicalensis radix*	Breast carbuncle	Heat flourishing, liver-qi stagnation	[74]

Wonders of Effective Prescription (Qi Xiao Liang Fang, Ming Dynasty, AD 1520)	*Forsythia suspense* decoction	*Citrus folium, Forsythia suspense Fructus, Liguszici, Trichosanthis semen, Gleditsiae Sinensis Spina, Citri reticulate viride pericarpium, Glycyrrhizae uralensis praparatae radix, Persicae semen*	Breast carbuncle Mammary node	Phlegm and blood stasis, liver-qi stagnation	[75]

Feng's Ace-Pack (Feng Shi Jin Nang, Qing Dynasty, AD 1722)	*Citrus forsythia* decoction	*Citrus folium, Citri reticulate viride pericarpium, Trichosanthis Fructus, Forsythia suspense Fructus, Persicae semen, Gleditsiae Sinensis Spina, Bulpleuri radix, Glycyrrhizae ureagenesis radix*	Breast carbuncle	Heat flaming in the beginning	[76]

Integration of Medicine (Yi Xue Ji Cheng, Qing Dynasty, AD 1873)	Dissipating nodules' decoction	*Citrus folium, Taraxaci cum radice herba, Cyperi rotundi rhizoma, Liguszici, Angelicae dahuricae radix, Fritillariae thunberii bulbus, Lonicerae japonicae flos, Perillae frutescentis folium, Alli fistulosi bulbus*	Mammary node	Heat flourishing, liver-qi stagnation	[77]

Bamboo Women's Treatment (Zhu Lin Nv Ke Zheng Zhi, Qing Dynasty, AD 1817)	*Lonicerae japonicae flos* decoction	*Citrus folium, Lonicerae japonicae flos, Astragali membranacei radix, Angelicae sinensis corpus radicis, Glycyrrhizae uralenisis radix*	Breast cancer	Vital qi deficiency and heat-induced toxicity	[73]

Orthodox Manual of External Medicine (Wai Ke Zheng Zong, Ming Dynasty, AD 1617)	*Citrus folium* powder	*Citrus folium, Bulpleuri radix, Citri reticulate pericarpium, Liguszici, Gardeniae jasminoidis Fructus, Citri reticulate viride pericarpium, Gypsum, Scutellariae baicalensis radix, Forsythia suspense Fructus, Glycyrrhizae uralensis praparatae radix*	Breast cancer	Heat flourishing, liver-qi stagnation	[54]

**Table 3:** The antitumor activity of NOB in BC.

Mechanism	Cell lines/animal models	*In vitro*/*in vivo*	Assay/treatment	Results	References
Cell viability inhibition	1 MCF-7 cell 2 Hs578T cells	*In vitro*	An MTT assay	(i) At 12.5 *μ*M, NOB decreased the cell viability to 85.3 ± 4.5% At 25 *μ*M, NOB decreased the cell viability to 79.3 ± 7.0% At 50 *μ*M, NOB decreased the cell viability to 71.2 ± 5.0% At 100 *μ*M, NOB decreased the cell viability to 60.3 ± 6.0% At 200 *μ*M, NOB decreased the cell viability to 54.4 ± 4.5% (ii) At 100 *μ*M, NOB showed greater toxicity toward the more invasive Hs578Ts(i)8 variant cell line, decreasing the cell viability to 51.6% after 72 h	[88] [89]

Cell growth inhibition	Hs578T cells	*In vitro*	Cell counting	After 24 h, limited effects were observed After 48 h, NOB reduced the number of cells by 40% After 72 h, NOB reduced the number of cells by 50%	[89]

Cell cycle arrest	Hs578T cells	*In vitro*	Western blot assay	Chk2 phosphorylation at T68 decreased after 10 min in the presence of NOB	[89]

Cell cycle arrest	MCF-7 cells	*In vitro*	Flow cytometry assay	At 100 *μ*M of NOB, a *G*1 block occurred in MCF-7 cells that was accompanied	[90]

Proliferation inhibition	MCF-7, T47D, MDA-MB-231c	*In vitro*	An MTT assay	NOB had a strong inhibitory effect on the proliferation of MDA-MB-231 cells and a weak inhibitory effect on the proliferation of HUVEC cells	[91]

Proliferation inhibition	MDA-MB-435, MCF-7	*In vitro*	Cell counting	Inhibition rates of NOB on treated cells ranged from 60 to 95%, beginning at 12 h and lasting up to 4 days, in all cell lines	[92]

Proliferation inhibition	Breast cancer model (i) Adult female Sprague Dawley rats with body weights (BWs) of 180–220 g (6-7 weeks old)	*In vivo*	DMBA (control rats) NOB (experimental rats)	(i) Compared with normal rats, the expression of proliferation-related proteins was increased in DMBA-treated rats (ii) NOB-treated rats showed downregulation of cell proliferation protein expression (iii) No significant changes were observed in the normal control group and rats treated with NOB alone	[93]

Apoptosis promotion	MCF-7 cells	*In vitro*	Flow cytometry assay	At 50 *μ*M, NOB induced cell apoptosis at the rates of 8.62 ± 3.5% At 100 *μ*M, NOB induced cell apoptosis at the rates of 11.2 ± 2.0% At 200 *μ*M, NOB induced cell apoptosis at the rates of 17.1 ± 3.7% The apoptosis rate of untreated cells was 5.4 ± 0.97%	[88]

Migration inhibition	1 MCF-7 cell 2 Hs578T cells	*In vitro*	Wound healing assay	(i) At 200 *μ*M of NOB, the inhibition rates of the migration were 38.2 ± 3.2% after 24 h At 200 *μ*M of NOB, the inhibition rates of the migration were 44.8 ± 2.5% after 48 h (ii) At 100 *μ*M of NOB, the migration was reduced by 40% in the Hs578Ts(i)8 cell	[88] [89]

Migration inhibition	MCF-7 cells	*In vitro*	Western blot assay	The expression levels of MMP-2 and MMP-9 were decreased in MCF-7 cell lines	[88]

Expression of protein connected with BC	MCF-7 cells	*In vitro*	Western blot and quantified protein levels of total p65 and nuclear p65 p38 and p-p38 Total Nrf2 and nuclear Nrf2	(i) At 50, 100, and 200 *μ*M, NOB downregulated the translocation of p65 in the MCF-7 cells (ii) NOB had no significant effect on p65 protein expression at this concentration (iii) At 100 and 200 *μ*M of NOB, the level of p-p38 was significantly increased At 50 *μ*M, NOB had no significant effect on p38 phosphorylation At 100 and 200 *μ*M, NOB significantly affected the expression of p38 (iv) At 100 and 200 *μ*M, NOB significantly reduced the levels of Nrf2 At 50 *μ*M, NOB had no obvious effect	[88]

Angiogenesis inhibition	MCF-7, T47D cells	*In vitro*	Angiogenesis assay Western blot assay RT-PCR analysis Electrophoretic mobility shift assay (EMSA)	At 200 *μ*M, NOB significantly inhibited tube formation in the extracellular matrix At 200 *μ*M of NOB, the inhibition of angiogenesis occurred in HUVEC cells (i) NOB downregulated the expression of phosphorylated EGFR (ii) NOB reduced the expression levels of phosphorylated Src, FAK, STAT3, and PXN (iii) After NOB treatment for 24 h, angiogenic factors were inhibited (iv) NOB downregulated the DNA/STAT3 complex in MCF-7 and T47D cells	[91]

## Abbreviations

NOB: Nobiletin
IARC: The International Agency for Research on Cancer
BC: Breast cancer
TNBC: Triple-negative breast cancer
EMT: Epithelial-mesenchymal transition
CNKI: China National Knowledge Infrastructure
UPLC: Ultrahigh-performance liquid chromatography
LC-ESI-MS/MS: Lipid chromatography combined with electrospray mass spectrometry
PMFs: Polymethoxyflavones
IUPAC: International Union of Pure and Applied Chemistry
5-DMN: 5-Demethylnobiletin
TCM: Traditional Chinese medicine
ROS: Reactive oxygen species
ER: Estrogen receptor
PR: Progesterone receptor
MTT: 3-(4,5-Dimethylthiazol-2)-2,5-diphenyltetrazolium bromide
CYP1A1: Cytochrome P4501A1
CYP1B1: Cytochrome P4501B1
MAPK: Mitogen-activated protein kinases
CXCR4: Chemokine (C-X-C motif) receptor 4
IC_50_: Half-maximal inhibitory concentration
ASD: Amorphous solid dispersion
Ras: GTP-binding protein
Raf: Serine-threonine protein kinase
MEK: Mitogen-activated protein kinase kinase
ERK1/2: Extracellular signal-regulated kinases
RTK: Receptor tyrosine kinase
GF: Growth factor
PI3K: Phosphoinositide 3-kinases
AKT: Protein kinase B
mTOR: Mammalian target of rapamycin
DMBA: 7,12-Dimethylbenz[a]anthracene
AP-1: Activator protein-1
PCNA: Proliferative cell nuclear antigen
NF-*κ*B: Nuclear factor kappa-B
MMP-2: Matrix metallopeptidase-2
MMP-9: Matrix metallopeptidase-9
EGF: Epidermal growth factor
EGFR: Epidermal growth factor receptor
FAK: Focal adhesion kinase
Src: Steroid receptor coactivator
STAT3: Signal transducer and activator of transcription 3
PXN: Paxillin, a focal adhesion-related protein
Nrf2: Nuclear factor erythroid 2-related factor 2
Bcl-2: B-cell lymphoma 2 protein.
